# Vertical stratification of sand fly diversity in relation to natural infections of *Leishmania* sp. and blood-meal sources in Jamari National Forest, Rondônia State, Brazil

**DOI:** 10.1186/s13071-020-04295-9

**Published:** 2020-08-17

**Authors:** Paula de Oliveira Leão, Antonio Marques Pereira Júnior, Paula Frassinetti Medeiros de Paulo, Luis Paulo Costa Carvalho, Ana Beatriz Nascimento Souza, Michelli Santos da Silva, Thaís Santos Castro, Moisés Thiago de Souza Freitas, Moreno Magalhães de Souza Rodrigues, Gabriel Eduardo Melim Ferreira, Jansen Fernandes Medeiros

**Affiliations:** 1 Fundação Oswaldo Cruz - Fiocruz Rondônia, Porto Velho, RO 76812-245 Brazil; 2 Programa de Pós Graduação em Biologia Experimental, Fundação Universidade Federal de Rondônia, Porto Velho, RO 76801-059 Brazil; 3 Instituto Nacional de Ciência e Tecnologia de Epidemiologia da Amazônia Ocidental - INCT-EpiAmO, Porto Velho, RO 76812-245 Brazil; 4 Departamento de Genética, Universidade Federal de Pernambuco, Recife, Pernambuco 50740-600 Brazil

**Keywords:** Zoonotic disease, Leishmaniasis, Vectors, Stratification, Canopy, Ground

## Abstract

**Background:**

Almost 1000 cases of American cutaneous leishmaniasis have been registered yearly in Rondônia State, Brazil. Little is known about the *Leishmania* transmission cycle (vectors and reservoirs) in the state. This study aimed to evaluate sand fly fauna from two vertical stratification layers in order to identify potential vectors and their blood-meal sources.

**Methods:**

The study was conducted in Jamari National Forest. Sand flies were collected in the canopy (15 m) and at ground level (1 m) using HP light traps during four months, February, April, August and October, 2018. Insects were identified to the species level, and females were subjected to DNA extraction and PCR targeting minicircle *k*DNA and *hsp*70 (for *Leishmania* detection and species identification), and *cytb* (to identify blood-meal sources). Exploratory data analysis was used to determine mean of abundance and species richness between stratifications. The *hsp*70 and *cytb* sequences were analyzed and compared with sequences from GenBank.

**Results:**

Overall, 68 species were identified from 15,457 individuals. On the Potosi trail, 7531 individuals of 49 species were collected; canopy captures totaled 6463 individuals of 46 species, while ground captures totaled 1068 individuals of 38 species. On the Santa Maria trail, 7926 individuals of 61 species were collected; canopy captures totaled 6136 individuals of 51 species, while ground captures totaled 1790 individuals of 53 species. A total of 23 pools were positive for *k*DNA (canopy *n* = 21, ground *n* = 2). Only two samples were sequenced for *hsp*70 (both in canopy); one sequence exhibited similarity with *Leishmania braziliensis* (*Lutzomyia davisi* pool) and another with *L. naiffi* (*Lu. antunesi* pool). The *cytb* fragment was amplified in 11 of 86 samples. Sample sequencing identified *cytb* DNA from 5 blood-meal sources: *Micrastur gilvicollis*, *Psophia viridis*, *Tamandua tetradactyla*, *Homo sapiens* and *Choloepus didactylus*.

**Conclusions:**

Sand fly fauna is more diverse in the canopy than at ground level. Factors such as blood-meal sources, resting sites, and abiotic components probably contribute to high abundance in the canopy. Our results reinforce the possibility that *Lu. antunesi* and *Lu*. *davisi* participate in *Leishmania* transmission in forest environments and may play an important role in transmission from sylvatic to human hosts.
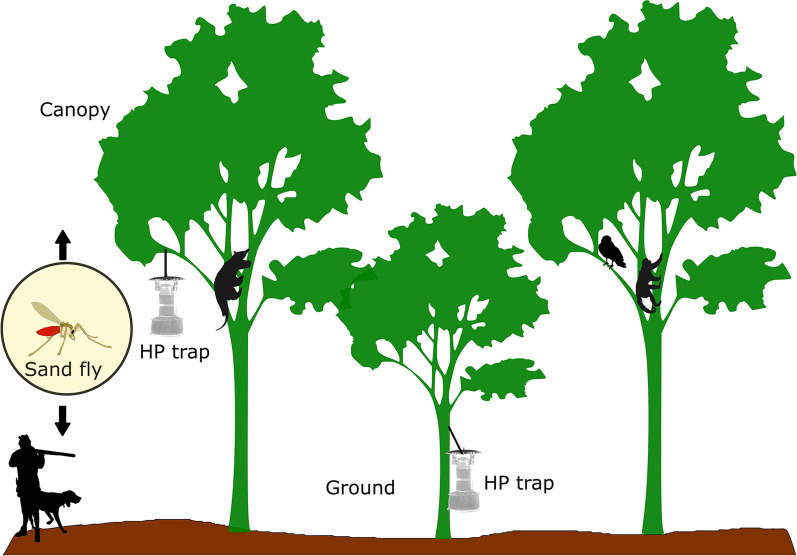

## Background

Phlebotomine sand flies (Diptera: Psychodidae: Phlebotominae) are small dipteran insects that play a role in the transmission of *Leishmania* species, the protozoans causing leishmaniases [1]. There are approximately 1000 phlebotomine sand fly species in the world; 286 occur in Brazil and 13 of these act as vectors [2].

Cutaneous leishmaniasis (CL) is characterized by localized lesions (LCL), but may also take the form of mucocutaneous leishmaniasis (ML), disseminated cutaneous leishmaniasis (DCL) or anergic diffuse cutaneous leishmaniasis (ADCL) [3]. This disease has a significant impact on public health; it is endemic in 98 countries with 0.7–1.2 million people infected per year and an estimated 350 million people at risk of contracting the disease [4]⁠. In 2018 alone, Brazil recorded approximately 17,000 cases of Leishmaniasis caused by seven *Leishmania* species: *Leishmania amazonensis*; *L. braziliensis* (the most prevalent); *L. guyanensis*; *L. lainsoni*; *L. lindenbergi*; *L. naiffi*; and *L. shawi* [5, 6]⁠.

In 2018, 1018 cases of CL were recorded in the state of Rondônia, Brazil [6]. CL has a zoonotic transmission cycle in which sand flies transmit *Leishmania* parasites between wild vertebrates. Humans are considered accidental hosts and most CL cases in Rondônia have been associated with anthropic activities (such as hunting, fishing, logging and mining) which are conducted in or near forest environments [7, 8]. Human cases of CL are caused by seven *Leishmania* species [8–10]⁠, and sand flies have been found carrying three of these species: *L*. *amazonensis*, *L*. *braziliensis* and *L*. *naiffi* [11–13]⁠.

To understand how CL is transmitted in the region, it is necessary to determine ecological parameters such as the composition and diversity of phlebotomine species (with a focus on vectors) and to identify which vertebrate blood meals participate in the maintenance of the *Leishmania* cycle in forest environments. In rainforests, the distribution of sand fly populations can be influenced by physical, biological and microclimatic conditions that differ between stratification levels [14]. For example, *Lutzomyia davisi* is found in abundance both in canopy and at ground level [14, 15], but *Lutzomyia flaviscutellata* tends to be distributed at ground level where it feeds primarily on rodents [16, 17].

Since 2016, our study group has been conducting surveys to assess vector species diversity and the prevalence of *Leishmania* parasites in Rondônia [12, 13, 18, 19]. To date, 143 sand fly species have been recorded in Rondônia [20]⁠ and *Lu*. *davisi* has been identified as a potential vector because it occurs in high abundance and field-collected females of the species have been tested positive for *Leishmania* DNA [10, 11, 13]. Few studies conducted in Rondônia have compared sand fly distribution patterns between stratification levels [12, 21] and little is known about *Leishmania* reservoirs in the region [10]⁠. In a previous study, we used molecular methods to target the *cytb* region and detected human, bovine and anteater DNA in sand flies collected from three different environments [13]⁠.

This study continues that line of research by describing the composition and distribution of sand fly species in two different stratifications (canopy and ground), and by aiming to: assess relative richness and abundance, detect natural infection with *Leishmania* DNA, and identify sand fly blood-meal sources in the natural environment.

## Methods

### Study area

**Fig. 1:**
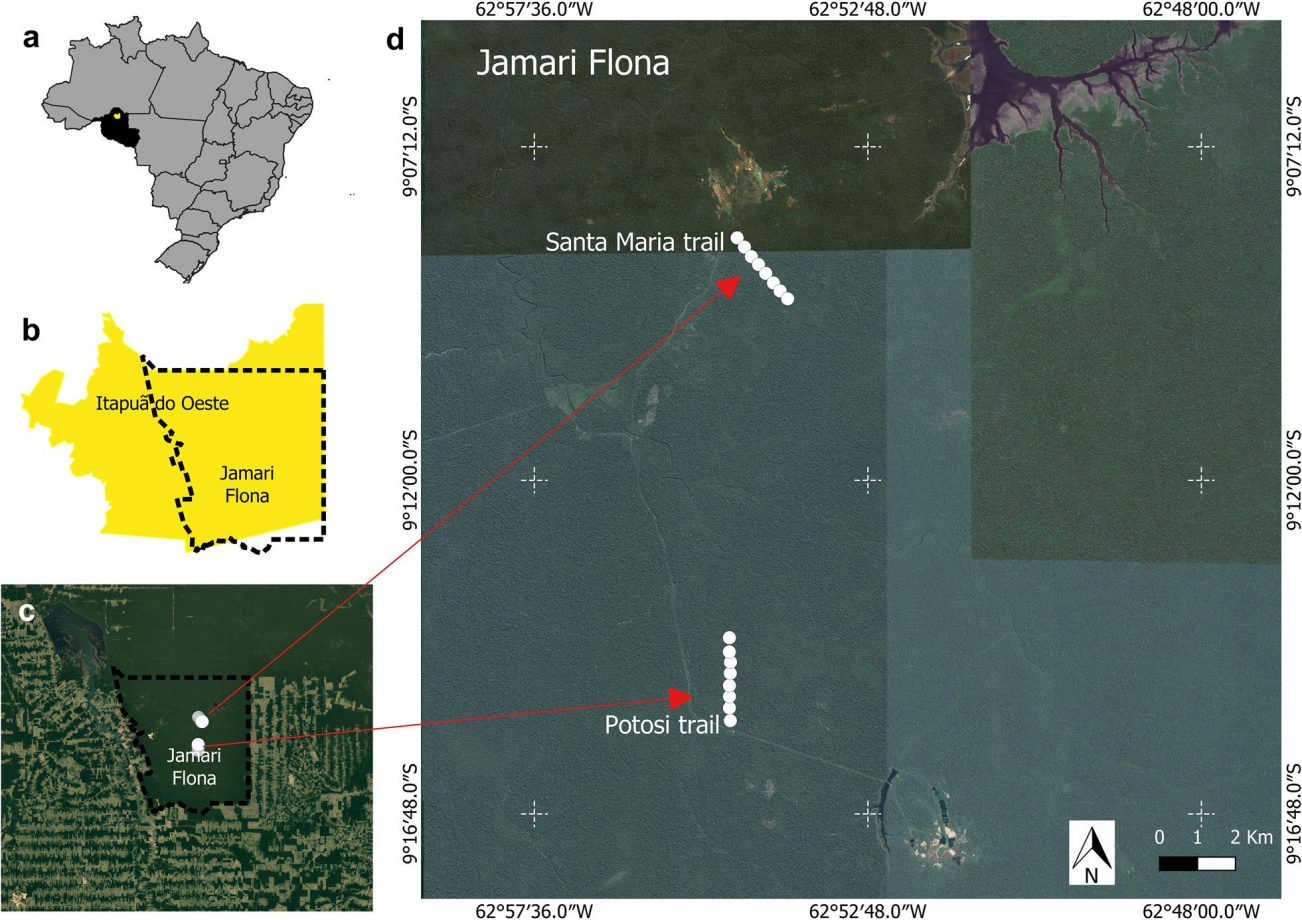
**a** Location of Itapuã do Oeste municipality in Rondônia State. **b** Jamari Flona territory within Itapuã do Oeste municipality. **c** Jamari Flona area with deforested area around. **d** The eight collection points selected on the Santa Maria and Potosi trails in Jamari Flona

Rondônia State is in the North Region of Brazil (Fig. 1a); it has an area of 237,576.2 km^2^, 52 municipalities and a population of 1,768,204. It has an equatorial dry and wet climate which generally entails a dry season between June and August and a rainy season between October and April, while May and September are months of seasonal transition. The average annual precipitation is 2000 mm; the relative humidity is 80–85%, and the average temperature 24 °C [22]⁠.

This study was performed in Jamari National Forest (Jamari Flona), located in northern Rondônia State, in the municipality of Itapuã do Oeste (Fig. 1b, c). This forest became a conservation unit on September 25, 1984. Jamari Flona has an area of approximately 225,000 ha [23]⁠. The phytophysiognomy is composed of 90% ombrophilous dense forest [24]⁠. Jamari Flona was a site of ore extraction in the 1950s. Part of this reserve has been designated as a Management Forest Unit (MFU) and part has been set aside for permanent preservation [23]⁠.

The Flona contains trails with 5 km in length used by the Chico Mendes Institute of Biodiversity Conservation (ICMBIO) under the National Programme of Biodiversity Knowledge. On the basis of yearly studies, we selected two trails for survey [12]⁠: the Potosi trail (09°15′36.14″S, 62°54′48.33″W) and the Santa Maria trail (09°08′22.65S, 62°54′49.04″W) (Fig. 1d).

Sand fly collection was authorized by ICMBIO under SISBIO system number 58563-3, and by the National System of Genetic Heritage Management and Associated Knowledge (SISGEN) under code AA32B8E, “Studies about Amazonian sand flies.”

### Sand fly collection and identification

Sand fly collections were performed for 5 consecutive nights in the months of February, April, August, and October, 2018. HP^®^ light traps [25] (Biomedica, Belo Horizonte, Brazil) were installed (from 18:00 to 7:00 h) at 8 points on the Santa Maria trail. The first point was positioned 500 m from the trailhead and the second point was positioned 300 m from the first point, and the remaining points were equally distanced from each other (at 300 m intervals) (Fig. 1d). At each point, traps were installed at ground level (1 m above ground) and in the canopy (15 m above ground); 16 HP traps were installed in total. In the canopy, traps were suspended from a “slingshot”, i.e. a fishing line (0.40 mm) weighted with a lead ball (20 g) and secured with a nylon rope (4 mm). The same design was replicated simultaneously on the Potosi trail. Mesh was not used in the HP light traps; instead, traps were outfitted with BD Falcon^TM^ 50 ml conical centrifuge tubes (BD Company, Bedford, USA) containing 96% ethanol.

After each night of collection, sand flies were separated from other insects using aspirators and, after chilling, examined under a binocular microscope at 5–20× magnification. Thereafter, the sand flies were stored in 96% ethanol, and maintained at a low temperature in loci. After each month of collection, specimens were placed in boxes containing ice in order to maintain a low temperature during transport to the Entomology Laboratory of Fiocruz Rondônia, Porto Velho. Males were mounted in Berleseʼs medium [26]⁠. The head and last 3 segments of each female were mounted, and the rest of the body was stored in 96% ethanol until molecular processing. Species identification was performed using the taxonomic keys by Young & Duncan [27], and Galati [28]. We adopted the nomenclatural of Young & Duncan [27] and the generic abbreviations proposed by Marcondes [29]⁠.

### Molecular detection of *Leishmania*

Non-engorged females of the most abundant species were sorted according to trail and stratification layer and separated into pools of 2–20 specimens. Pools were subjected to DNA extraction and PCR assays targeting minicircle *k*DNA using the primers 5′-GGG (G/T)AG GGG CGT TCT (G/C)CG AA-3′ and 5′-(G/C)(G/C)(G/C) (A/T)CT AT(A/T) TTA CAC CAA CCC C-3′ (120 bp). Positive samples from the first reaction were subjected to PCR targeting the *hsp*70 region using the primers 5′-GGA CGA GAT CGA GCG CAT GGT-3′ and 5′-TCC TTC GAC GCC TCC TGG TTG-3′ (240 bp), as described elsewhere [12, 30]. *Lutzomyia ubiquitalis* males and the *L*. *amazonensis* reference strain IOC/L0575 (IFLA/BR/1967/PH8) were used as the positive controls and ultrapure water was used as the negative control.

### Blood-meal identification

Engorged females were separated according to species, trail and strata. Three samples were used as the negative control during DNA extraction:1 sample containing DNA-free water and 2 samples containing a female sand fly with no blood present in the gut. DNA extraction followed the phenol/chloroform protocol described by Sambrook & Russell [31]⁠. PCR used the primers *cytb* 1 and *cytb* 2, which are complementary to the conserved region of the cytochrome *b* gene in vertebrate mtDNA [32]⁠.

The amplification reactions (total volume of 50 µl) contained 25 µl (1×) Go Taq Colorless (Promega, Madison, USA), 1.5 µl of each primer (*cytb* 1 and *cytb* 2, 10 µM) and 5 µl of DNA (< 250 ng). Amplifications were performed in a thermocycler (Veriti^®^; Applied Biosystems, Foster City, USA) with an initial denaturation of 95 °C for 5 min, followed by 35 cycles of denaturation at 95 °C for 30 s, annealing at 53 °C for 30 s and extension at 72 °C for 1 min, with a final extension step at 72 °C for 6 min. Amplified products were purified using the QIAquick Purification Kit (Qiagen, Hilden, Germany) and submitted to the Fiocruz Sequencing Facility (RTP01E, Belo Horizonte, Brazil)

### Data analysis

Exploratory data analysis (EDA) was used to describe the abundance and total mean richness of the species collected between strata (canopy × ground). Mean abundance and mean species richness were computed per trap per night. Minimum infection rates were obtained by multiplying the number of *k*DNA positive pools by 100 and dividing the result by the total number of females in these pools [12]. The proportion of engorged to non-engorged females was determined for the most abundant species from each stratification. All data were analyzed using the R statistical environment [33] (R scripts are described in Additional file 1: Dataset S1, Additional file 2: Dataset S2)⁠.

The *hsp*70 and *cytb* genes were sequenced in duplicate for each sample and the sequences obtained were assembled and analyzed using the *Staden* package [34]⁠ based on Phred values of 30 or above [35]⁠. The consensus sequences were submitted to a BLASTn search (http://blast.ncbi.nlm.nih.gov/Blast.cgi) and compared with sequences from GenBank (http://www.ncbi.nlm.nih.gov/genbank/). All new sequences generated in this study were deposited in the GenBank database under the accession numbers MT234139, MT260076-MT7260081, MT293514-MT7293518 and MT300189.

## Results

A total of 15,457 individuals comprising 68 species were collected. Some females were identified only at the subgenus level: *Lutzomyia* (*Trichophoromyia*) sp. (*n* = 30) and *Lutzomyia* (*Trichopygomyia*) sp. (*n* = 339). The most abundant species were *Lutzomyia antunesi* (*n* = 2530), *Lu. ayrozai* (*n* = 2198), *Lu. davisi* (*n* = 2019), *Lu. yuilli yuilli* (*n* = 1483) and *Lu*. *ubiquitalis* (*n* = 1153) (Table 1). The sex ratio of the potential vectors is outlined in Table 2.

**Table 1:** Sand flies collected from canopy and ground strata on two trails in Jamari Flona, located in Itapuã do Oeste municipality, Rondônia State

Species	Potosi		Santa Maria		Total	%
	Canopy	Ground	Canopy	Ground		
*Brumptomyia brumpti*	1^b^	–	–	–	1	0.01
*Lutzomyia* (*Evandromyia*) *infraspinosa*	–	–	1^d^	–	1	0.01
*Lutzomyia* (*Evandromyia*) sp.	–	–	1	–	1	0.01
*Lutzomyia* (*Evandromyia*) *tarapacaensis*	27	171	4	75	277	1.79
*Lutzomyia* (*Lutzomyia*) *flabellata*	–	–	5^d^	–	5	0.03
*Lutzomyia* (*Lutzomyia*) *sherlocki*	28	31	39	39	137	0.89
*Lutzomyia* (*Nyssomyia*) *anduzei*^a^	1^b^	–	–	–	1	0.01
*Lutzomyia* (*Nyssomyia*) *antunesi*^a^	1873	78	530	49	2530	16.37
*Lutzomyia* (*Nyssomyia*) *flaviscutellata*^a^	2	6	17	9	34	0.22
*Lutzomyia* (*Nyssomyia*) *richardwardi*	31	12	136	39	218	1.41
*Lutzomyia* (*Nyssomyia*) *shawi*	–	–	–	1^e^	1	0.01
*Lutzomyia* (*Nyssomyia*) sp.	2	–	4	2	8	0.05
*Lutzomyia* (*Nyssomyia*) *umbratilis*^a^	163	28	49	10	250	1.62
*Lutzomyia* (*Nyssomyia*) *whitmani*^a^	4	2	27	5	38	0.25
*Lutzomyia INyssomyia*) *yuilli yuilli*	848	79	531	25	1483	9.59
*Lutzomyia* (*Pressatia*) *calcarata*	–	–	1^d^	–	1	0.01
*Lutzomyia* (*Pressatia*) sp.	–	–	–	1	1	0.01
*Lutzomyia* (*Pressatia*) *triacantha*	1^b^	–	–	–	1	0.01
*Lutzomyia* (*Psathyromyia*) *abbonenci*	1^b^	–	–	–	1	0.01
*Lutzomyia* (*Psathyromyia*) *bigeniculata*	1	2	9	1	13	0.08
*Lutzomyia* (*Psathyromyia*) *campbelli*	4^b^	–	1^d^	–	5	0.03
*Lutzomyia* (*Psathyromyia*) *dendrophyla*	4	4	20	3	31	0.20
*Lutzomyia* (*Psathyromyia*) *lutziana*	1^b^	–	3	1	5	0.03
*Lutzomyia* (*Psathyromyia*) *punctigeniculata*	1	1	–	–	2	0.01
*Lutzomyia* (*Psathyromyia*) *scaffi*	–	–	3^d^	–	3	0.02
*Lutzomyia* (*Psathyromyia*) sp.	–	–	–	1	1	0.01
*Lutzomyia* (*Psathyromyia*) sp. (Shannoni series)	1	–	1	–	2	0.01
*Lutzomyia* (*Psychodopygus*) *amazonensis*	2	1	35	25	63	0.41
*Lutzomyia* (*Psychodopygus*) *ayrozai*^a^	1256	110	786	46	2198	14.22
*Lutzomyia* (*Psychodopygus*) *bispinosa*	46	1	45	2	94	0.61
*Lutzomyia* (*Psychodopygus*) *carrerai carrerai*^a^	53	7	226	11	297	1.92
*Lutzomyia* (*Psychodopygus*) *chagasi*	47	17	51	6	121	0.78
*Lutzomyia* (*Psychodopygus*) *claustrei*	11^b^	–	26	2	39	0.25
*Lutzomyia* (*Psychodopygus*) *complexa*^a^	2^b^	–	5	1	8	0.05
*Lutzomyia* (*Psychodopygus*) *davisi*^a^	521	121	1225	152	2019	13.06
*Lutzomyia* (*Psychodopygus*) *geniculata*	221	31	160	38	450	2.91
*Lutzomyia* (*Psychodopygus*) *hirsuta hirsuta*^a^	10	1	133	10	154	1.00
*Lutzomyia* (*Psychodopygus*) *lainsoni*	21	10	35	64	130	0.84
*Lutzomyia* (*Psychodopygus*) *leonidasdeanei*	1^b^	–	–	–	1	0.01
*Lutzomyia* (*Psychodopygus*) *llanosmartinsi*	13	1	80	1	95	0.61
*Lutzomyia* (*Psychodopygus*) sp.	11	2	28	2	43	0.28
*Lutzomyia* (*Psychodopygus*) sp. (Chagasi series)	609	28	468	14	1119	7.24
*Lutzomyia* (*Sciopemyia*) *fluviatilis*	4	3	–	4^e^	11	0.07
*Lutzomyia* (*Sciopemyia*) *servulolimai*	–	2^c^	–	–	2	0.01
*Lutzomyia* (*Sciopemyia*) *sordellii*	25	25	15	49	114	0.74
*Lutzomyia* (*Sciopemyia*) sp.	1	–	2	2	5	0.03
*Lutzomyia* (*Trichophoromyia*) *auraensis*^a^	–	2^c^	2	2	6	0.04
*Lutzomyia* (*Trichophoromyia*) *clitella*	–	–	9	46	55	0.36
*Lutzomyia* (*Trichophoromyia*) *flochi*	–	–	1^d^	–	1	0.01
*Lutzomyia* (*Trichophoromyia*) *loretonensis*	–	–	–	1^e^	1	0.01
*Lutzomyia* (*Trichophoromyia*) sp.	1	–	16	13	30	0.19
*Lutzomyia* (*Trichophoromyia*) *ubiquitalis*^a^	109	132	132	780	1153	7.46
*Lutzomyia* (*Trichopygomyia*) *dasypodogeton*	144	41	72	15	272	1.76
*Lutzomyia* (*Trichopygomyia*) *longispina*	–	–	–	1^e^	1	0.01
*Lutzomyia* (*Trichopygomyia*) *rondoniensis*	67	14	12	5	98	0.63
*Lutzomyia* (*Trichopygomyia*) sp.	167	43	94	35	339	2.19
*Lutzomyia* (*Trichopygomyia*) *wagleyi*	29	9	5	1	44	0.28
*Lutzomyia* (*Viannamyia*) *furcate*	20	16	764	39	839	5.43
*Lutzomyia* (*Viannamyia*) *tuberculata*	38	17	204	15	274	1.77
*Lutzomyia aragaoi* (Aragaoi Group)	20	8	29	11	68	0.44
*Lutzomyia dreisbachi* (Dreisbachi Group)	–	–	5	1	6	0.04
*Lutzomyia andersoni* (Migonei Group)	–	–	–	1^e^	1	0.01
*Lutzomyia apurinan* (Migonei Group)	1	1	1	6	9	0.06
*Lutzomyia bacula* (Migonei Group)	1	2	2	6	11	0.07
*Lutzomyia migonei* (Migonei Group)^a^	–	–	22	4	26	0.17
*Lutzomyia monstruosa* (Migonei Group)	–	–	–	2^e^	2	0.01
*Lutzomyia sericea* (Migonei Group)	–	–	–	1^e^	1	0.01
*Lutzomyia walkeri* (Migonei Group)	1^b^	–	–	1^e^	2	0.01
*Lutzomyia williamsi* (Migonei Group)	–	–	2	1	3	0.02
*Lutzomyia termitophila* (Migonei Group)	1	3	4	8	16	0.10
*Lutzomyia peresi* (Oswaldoi Group)	–	–	–	2^e^	2	0.01
*Lutzomyia rorotaensis* (Oswaldoi Group)	–	4^c^	13	5	22	0.14
*Lutzomyia villelai* (Oswaldoi Group)	–	–	–	5^e^	5	0.03
*Lutzomyia saulensis* (Saulensis Group)	–	–	1^d^	–	1	0.01
*Lutzomyia wilsoni* (Saulensis Group)	9	1	22	88	120	0.78
*Lutzomyia duckei* (Verrucarum Group)	–	–	8^d^	–	8	0.05
*Lutzomyia fiocruzi* (Verrucarum Group)	6	1	13	4	24	0.16
*Lutzomyia serrana* (Verrucarum Group)	1^b^	–	1	1	3	0.02
Total	6463	1068	6136	1790	15,457	100

^a^Potential vectors according Rangel [2]

^b^Species occur only in canopy (Potosi trail)

^c^Species only at the ground level (Potosi trail)

^d^Species occur only in canopy (Santa Maria trail)

^e^Species only at the ground level (Santa Maria)

**Table 2:** Abundance of females (♀) and males (♂) and sex ratio of potential vector species captured in the canopy and at the ground level in Jamari Flona, municipality of Itapuã do Oeste

Species	Potosi						Santa Maria					
	Canopy		(♀:♂)	Ground		(♀:♂)	Canopy		(♀:♂)	Ground		(♀:♂)
	♀	♂		♀	♂		♀	♂		♀	♂	
*Lutzomyia anduzei*	1	–	–	–	–	–	–	–	–	–	–	–
*Lutzomyia antunesi*	526	1347	0.4:1	34	44	0.7:1	320	210	1.5:1	36	13	2.7:1
*Lutzomyia flaviscutellata*	2	–	–	3	3	1:1	10	7	1.4:1	4	5	0.8:1
*Lutzomyia umbratilis*	141	22	6.4:1	28	–	–	32	17	1.8:1	9	1	9:1
*Lutzomyia whitmani*	1	3	0.3:1	2	–	–	16	11	1.4:1	5	–	–
*Lutzomyia yuilli yuilli*	770	78	9.8:1	72	7	10.3:1	480	51	9.4:1	19	6	3.1:1
*Lutzomyia ayrozai*	818	438	1.8:1	36	74	0.4:1	478	308	1.5:1	24	22	1.1:1
*Lutzomyia carrerai carrerai*	46	7	6.5:1	4	3	1.3:1	162	64	2.5:1	10	1	1:1
*Lutzomyia complexa*	–	2	–	–	–	–	–	5	–	–	1	–
*Lutzomyia davisi*	321	200	1.6:1	54	67	0.8:1	777	448	1.7:1	83	69	1.2:1
*Lutzomyia hirsuta hirsuta*	10	–	–	1	–	–	97	36	2.7:1	6	4	1.5:1
*Lutzomyia auraensis*	–	–	–	–	2	–	–	2	–	–	2	–
*Lutzomyia ubiquitalis*	39	70	0.5:1	16	116	0.1:1	37	95	0.4:1	115	665	0.1:1
*Lutzomyia migonei*	–	–	–	–	–	–	7	15	0.4:1	4	–	–

### Diversity between stratifications

On the Potosi trail, 7531 individuals were collected, and 49 species were identified. Eleven species were recorded solely in canopy, 3 species were recorded solely at ground level, and 34 species were collected in both strata (Table 1).

**Fig. 2:**
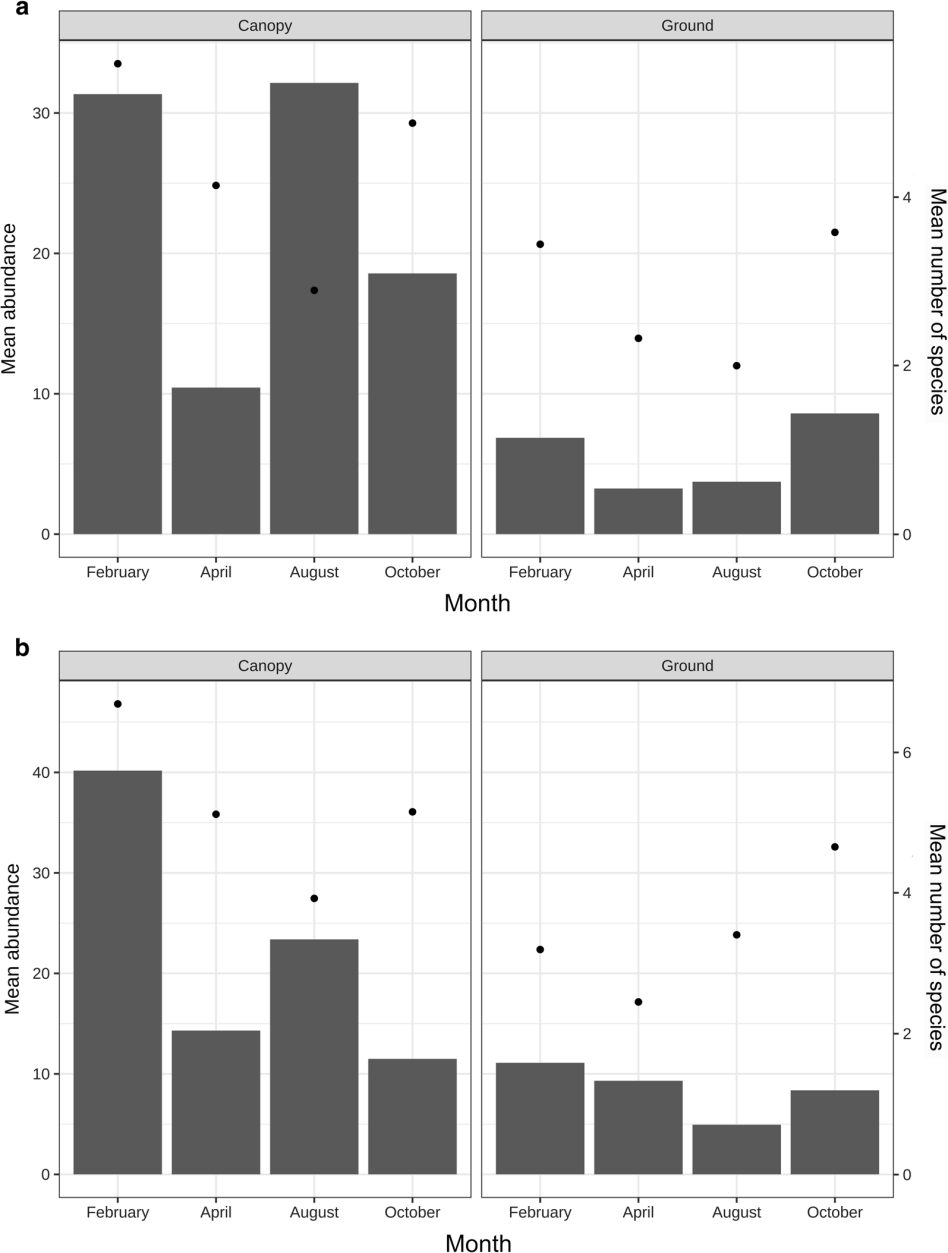
Mean of abundance (bars) and species richness (circles) of sand flies captured per trap/night in canopy and at the ground level of the Potosi trail (**a**) and the Santa Maria trail (**b**), located in Jamari Flona, Itapuã do Oeste municipality

In the canopy 6463 individuals were collected, and 46 species were identified. In the canopy, the highest mean abundance per trap/night occurred in August (32.1 individuals), followed by February (31.3), October (18.6) and April (10.4). The highest mean richness captured per trap/night occurred in February (5.6 species), followed by October (4.9), April (4.1) and August (2.9) (Fig. 2a). The most abundant species in the canopy were *Lu. antunesi* (1873), *Lu. ayrozai* (1256), *Lu. y. yuilli* (848) and *Lu*. *davisi* (521) (Table 1).

At the ground level, 1068 individuals were collected, and 38 species were identified. The highest mean abundance per trap/night occurred in October (8.6 individuals), followed by February (6.9), August (3.7) and April (3.3). The highest mean richness captured per trap/night occurred in October (3.6 species), followed by February (3.4), April (2.3) and August (2.0) (Fig. 2a). The most abundant species at ground level were *Lu. tarapacaensis* (171), *Lu*. *ubiquitalis* (132), *Lu. davisi* (121) and *Lu. ayrozai* (110).

On the Santa Maria trail, 7926 individuals were collected, and 61 species were identified. Eight species were recorded solely in canopy, 10 species were recorded solely at ground level, and 43 species were collected in both strata (Table 1).

In the canopy, 6136 individuals were collected, and 51 species were identified. The highest mean abundance per trap/night occurred in February (40.2 individuals), followed by August (23.4), April (14.3) and October (11.5). The highest mean richness captured per trap/night occurred in February (6.7 species), followed by October (5.2), April (5.1) and August (3.9) (Fig. 2b). The most abundant species in the canopy were *Lu*. *davisi* (1225), *Lu. ayrozai* (786), *Lu. furcata* (764), *Lu. y. yuilli* (531), and *Lu. antunesi* (530) (Table 1).

At the ground level, 1790 individuals were collected, and 53 species were identified. The highest mean abundance occurred in February (11.1 individuals), followed by April (9.3), October (8.4) and August (4.9). The highest mean richness occurred in October (4.7 species), followed by August (3.4), February (3.2) and April (2.5) (Fig. 2b). The most abundant species at ground level was *Lu*. *ubiquitalis* (780).

### Detection of *Leishmania* DNA

A total of 2817 sand fly females were sorted into 194 pools (Table 2): 67 pools from Santa Maria trail (canopy: 52 pools; ground: 15 pools) and 127 pools from Potosi trail (canopy: 105 pools; ground: 22 pools). The 120-bp minicircle *k*DNA fragment was amplified in 23 pools: 6 from Santa Maria trail (all from canopy) and 17 from Potosi trail (canopy: 15; ground: 2). The minimal infection rate was 0.81% (23/2817). The 240 bp *hsp*70 fragment was amplified in 8 pools: 5 pools from the Santa Maria trial and 3 pools from the Potosi trail. Sequencing was successful for 2 samples in which the *hsp*70 fragment was amplified. Both samples were from the canopy level of the Santa Maria trail; 1 sequence exhibited similarity with *L. braziliensis* (*Lu*. *davisi* pool) and the other exhibited similarity with *L. naiffi* (*Lu. antunesi* pool) (Table 3).

**Table 3:** Separation of sand fly species into pools for *Leishmania k*DNA detection

Species	Potosi		Santa Maria		Pools	♀
	Canopy	Ground	Canopy	Ground		
*Lutzomyia* (*Evandromyia*) *tarapacaensis*	–	1	–	1	2	17
*Lutzomyia* (*Nyssomyia*) *antunesi*	15 (2)	2	12 (2^a^)	3	32 (4)	502
*Lutzomyia* (*Nyssomyia*) *richardwardi*	1	1	1	–	3	24
*Lutzomyia* (*Nyssomyia*) *umbratilis*	4 (1)	2	2	–	8 (1)	67
*Lutzomyia* (*Nyssomyia*) *whitmani*	–	1	1	–	2	5
*Lutzomyia* (*Nyssomyia*) *yuilli yuilli*	27 (4)	2	8 (1)	1	38 (5)	642
*Lutzomyia fiocruzi* (Verrucarum Group)	–	–	1	–	1	3
*Lutzomyia* (*Psychodopygus*) *ayrozai*	35 (2)	4	5	1	45 (2)	829
*Lutzomyia* (*Psychodopygus*) *bispinosa*	1	–	1	–	2	15
*Lutzomyia* (*Psychodopygus*) *c. carrerai*	1	–	1	–	2	8
*Lutzomyia* (*Psychodopygus*) *claustrei*	–	–	1	1	2	7
*Lutzomyia* (*Psychodopygus*) sp. (Chagasi series)	8	3	3	1	15	222
*Lutzomyia* (*Psychodopygus*) *davisi*	11 (6)	4 (2)	12 (2^a^)	5	32 (10)	394
*Lutzomyia* (*Psychodopygus*) *geniculata*	1	–	1	–	2	27
*Lutzomyia* (*Psychodopygus*) *h. hirsuta*	–	–	2 (1)	–	2 (1)	25
*Lutzomyia* (*Trichophoromyia*) *ubiquitalis*	1	2	1	2	6	30
Total	105 (15)	22 (2)	52 (6)	15	194 (23)	2817

^a^*hsp*70 region was identified in this species

*Notes*: Numbers in parentheses correspond to pools of sand fly species that tested positive for *k*DNA from *Leishmania* spp.

### Blood-meal identification

**Fig. 3:**
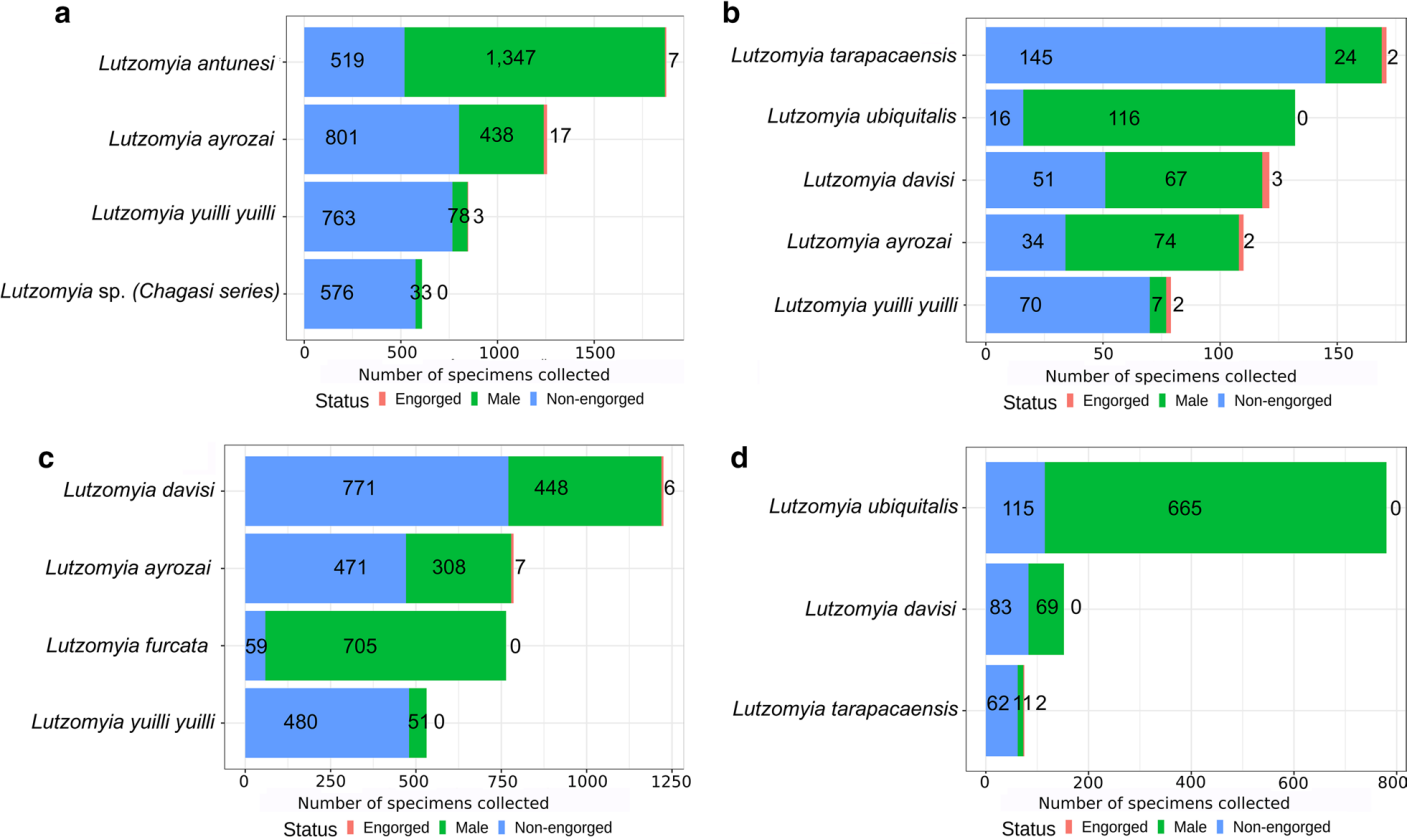
Proportion of engorged to non-engorged females and males from the most abundant species in the canopy (**a**) and at the ground level (**b**) of the Potosi trail, and the canopy (**c**) and the ground (**d**) of Santa Maria trail, Jamari Flona, Itapuã do Oeste municipality

Of the 8788 females collected 86 (0.97%) were engorged: 28 from the Santa Maria trail (canopy: 22; ground: 6) and 58 from the Potosi trail (canopy: 45; ground: 13). The most abundant species with blood present in the gut were *Lu*. *ayrozai* (*n* = 24), *Lu*. *antunesi* (*n* = 7) and *Lu*. *davisi* (*n* = 6) from canopy (Fig. 3a–c) and *Lu*. *davisi* (*n* = 3), *Lu. tarapacaensis* (*n* = 3), *Lu*. *ayrozai* (*n* = 2), *Lu*. *antunesi* (*n* = 2) and *Lu*. *y*. *yuilli* (*n* = 2) from the ground level (Fig. 3b–d).

Amplification of the *cytb* region was attempted for all 86 samples and successful identification was achieved for 11 individuals (0.12%): *Lu. antunesi* (*n* = 4); *Lutzomyia fiocruzi* (*n* = 2); *Lu. ayrozai* (*n* = 2); and *Lu. davisi* (*n* = 3). The sequenced amplification products exhibited similarity with the *cytb* DNA of anteaters (*Tamandua tetradactyla*), humans (*Homo sapiens*), sloths (*Choloepus didactylus*) and two species of bird: the lined forest falcon (*Micrastur gilvicollis*); and the green-winged trumpeter (*Psophia viridis*) (Table 4).

**Table 4:** Sequencing results for *hsp*70 and *cytb* gene fragments obtained from sand flies collected in Jamari FLONA, Itapuã do Oeste, Rondônia

Species	Primer	GenBank ID	Sand fly species	Strata (Trail)	Score	Query cover (%)	E-value	Identity (%)
*Leishmania braziliensis*	*hsp70*	GU071180.1	*Lutzomyia davisi*	Canopy (SM)	331	98	5.00E−87	98
*Leishmania naiffi*	*hsp70*	FN395056.2	*Lutzomyia antunesi*	Canopy (SM)	320	100	1.00E−83	97
*Choloepus didactylus*	*cytb*	KR336792.1	*Lutzomyia fiocruzi*	Canopy (PT)	440	98	1.00E−119	94
*Micrastur gilvicollis*	*cytb*	DQ780881.1	*Lutzomyia fiocruzi*	Canopy (SM)	438	100	5.00E−119	94
*Homo sapiens*	*cytb*	LC088152.1	*Lutzomyia ayrozai*	Canopy (PT)	542	99	3.00E−150	100
*Homo sapiens*	*cytb*	LC088152.1	*Lutzomyia davisi*	Ground (PT)	536	100	2.00E−148	99
*Tamandua tetradactyla*	*cytb*	KT818552.1	*Lutzomyia antunesi*	Canopy (PT)	525	100	3.00E−145	100
*Tamandua tetradactyla*	*cytb*	KT818552.1	*Lutzomyia antunesi*	Canopy (PT)	534	97	6.00E−148	99
*Tamandua tetradactyla*	*cytb*	KT818552.1	*Lutzomyia antunesi*	Canopy (PT)	521	100	4.00E−144	100
*Tamandua tetradactyla*	*cytb*	KT818552.1	*Lutzomyia antunesi*	Canopy (PT)	523	100	1.00E−144	99
*Tamandua tetradactyla*	*cytb*	KT818552.1	*Lutzomyia ayrozai*	Canopy (PT)	525	98	3.00E−145	99
*Tamandua tetradactyla*	*cytb*	KT818552.1	*Lutzomyia davisi*	Canopy (SM)	521	98	4.00E−144	99
*Psophia viridis*	*cytb*	DQ485901.1	*Lutzomyia davisi*	Canopy (SM)	520	99	2.00E−143	98

*Abbreviations*: PT, Potosi trail; SM, Santa Maria trail

## Discussion

We evaluated the abundance and richness patterns of sand fly fauna in two stratifications in Jamari Flona, Rondônia, Brazil. Abundance was highest in the canopy, where approximately 60% of individuals belonged to four species, *Lu*. *antunesi*, *Lu*. *y. yuilli*, *Lu*. *ayrozai* and *Lu*. *davisi*. These findings corroborate those of Resadore et al. [12]⁠ who surveyed the same locality and found *Lu*. *y. yuilli* and *Lu*. *davisi* to be the most abundant species in the canopy; however, at ground level we found *Lu*. *ubiquitalis* to be the most abundant species while Resadore et al. [12] found this species in low abundance in both strata. Souza et al. [36] also found *Lu*. *ubiquitalis* in abundance at ground level, which may indicate that the distribution of this species is specific to ground level habitats.

For each month of collection on both trails, the mean abundance and richness of sand flies per trap/night was higher in the canopy than at ground level. This finding corroborates the findings of other Amazonian studies [12, 14, 16]. The canopy provides more plentiful resting sites among leaves and trunks, and offers a greater variety of nocturnal blood-meal sources which may attract sand flies since sand flies are most active at night. Both factors, blood meals and resting sites, may contribute to the presence of sand flies in the upper stratum [17].

Overall, abundance was highest in February and October, which is typical since sand flies tend to be captured mostly during the rainy season [17, 36, 37]. In Rondônia, the rainy season generally begins in October and peaks between January and April [22]. In the Central Amazon, between 1977 and 1978, Arias & Freitas [17] observed the highest number of sand fly captures in November, December and May, but these authors speculated that the high number of captures may have resulted from a high rate of adult emergence among *Lu*. *anduzei* and *Lu*. *umbratilis*.

We were surprised to find that sand fly abundance was higher in August than in October and April in the canopy of the Potosi trail, even though August is part of the dry season. Godoy et al. [37] verified a negative correlation between precipitation and the abundance of the *Lu. antunesi* in the municipality of Guaraí, Tocantins State; thereby demonstrating that precipitation can influence the abundance of sand fly populations. Our study did not account for abiotic variables and our sample design did not allow us to evaluate the impact of seasonality; therefore, it was difficult to attribute a cause to the high level of sand fly abundance observed in the dry month of August. Further studies will need to determine which factors influenced the capture rates that we observed.

The presence of *Leishmania* species and the identity of blood-meal sources were determined for sand flies from both strata. For *Leishmania* detection, of the 23 pools that were positive for minicircle *k*DNA and the eight pools in which *hsp*70 was amplified, only two samples were successfully identified by sequencing. The *k*DNA region has a high number of copies and is more sensitive to amplification, but it is present in the mitochondrial DNA of the family Trypanosomatidae and thus does not permit specific identification of *Leishmania*; the *hsp*70 region does permit specific identification of *Leishmania* species, but it has fewer copies and is less sensitive to amplification [30]. In this study, the identification of six *hsp*70-positive samples was not possible due to the low quality of the sequences.

The *Leishmania* species identified here are *L*. *braziliensis* and *L*. *naiffi*. Currently in Rondônia, *L*. *braziliensis* is the most prevalent species of *Leishmania* associated with human cases of leishmaniasis and it has been found previously in sand flies [10, 11, 13]. *Leishmania braziliensis* has a variety of hosts [38]; thus, different sand fly species with different blood-meal sources could nevertheless acquire the same parasite from a variety of vertebrate hosts, which may account for the presence of *L*. *braziliensis* in many sand fly species. We found *L*. *braziliensis* DNA in *Lu*. *davisi*, which corroborates the findings of other studies conducted in Rondônia [10, 13]⁠ and reinforces the possibility that *Lu*. *davisi* is a putative vector of *L*. *braziliensis*.

*Leishmania naiffi* is uncommon in human cases but some studies indicate the possibility that *L. naiffi* infection has been underreported in the North Region of Brazil [39]⁠. The natural host of *L. naiffi* is *Dasypus novemcinctus* [40]. In humans, infection with *L. naiffi* generally manifests as localized lesions that are amenable to spontaneous cure or rapid treatment [39, 41–43]⁠. *Leishmania naiffi* has been identified in only one human case of leishmaniasis in Rondônia, a 35 year-old man from the municipality of Rolim de Moura [43]⁠.

In Rondônia, *L. naiffi* has been found in field-collected females of the *Lu*. *ayrozai* [5]⁠, also females of *Lu*. *davisi* and *Lu. hirsuta hirsuta* have been found carrying *L*. *naiffi* flagellates [10, 11]⁠. In this study, no *Lu*. *ayrozai* females were found with *Leishmania* DNA, but this is the first time that *L*. *naiffi* has been identified in *Lu*. (*Nyssomyia*) sand flies from Rondônia. *Lutzomyia antunesi* is abundant in many parts of the state and may act as a vector there [11, 13, 19]⁠; however, to date, *Lu*. *antunesi* is a proven vector only of *L. lindenbergi* in Pará State [44]⁠. Given that *Lu*. *davisi* and *Lu*. *antunesi* are frequently found in abundance in Rondônia, further studies need to examine colonization patterns and the vector competence and capacity of these species in relation to *Leishmania* transmission cycles.

Of the 8788 females collected, 86 were engorged (less than 1% of the total). This proportion is in line with two previous studies, one in which 15 out of 4089 females were engorged [13]⁠, and another in which seven out of 708 females were engorged [45]. These extremely small proportions suggest that light traps are not an effective method for collecting engorged females.

Blood-meal identification was possible for only 0.12% of total females captured. This decrease (from 1% to 0.12%) may be due to differing stages of digestion among engorged females. After blood-feeding, the blood meal is generally directed to the midgut where enzymatic activity increases, and this step may degrade the DNA and thus reduce PCR sensitivity [46]⁠. For example, in one study the efficacy of amplification was reduced by 20% in mosquito samples that were submitted to PCR (targeting the *cytb* gene) 33 h after blood-feeding [47]⁠, and, in another study, Baum et al. [48]⁠ subjected blood from 93 engorged females to PCR targeting the PNOC partial gene 24 h after blood-feeding, and blood-meal identification was possible in only 27 females. These observations demonstrate that more effective technologies need to be developed for the detection of blood-meal sources.

Our findings improve the knowledge of sand fly blood-meal sources in sylvatic environments. The abundance of *Lu*. *antunesi*, *Lu*. *ayrozai* and *Lu*. *davisi* in the canopy may indicate that these species blood-feed on arboreal animals. In a study conducted in the Central Amazon, Arias et al. [17] found that when a natural host is ground-dwelling, the associated sand fly species feed at ground level, and when the natural host is canopy-dwelling the associated species feed at canopy level. However, given the low sample size obtained in our collections and given that anteaters, sloths, and birds frequently move between canopy and ground level, it was not possible to determine whether or not feeding was occurring arboreally. Similarly, although human DNA was found in a *Lu*. *davisi* female collected in the canopy, it was impossible to determine if this species favors blood meals from a specific stratum. We believe that this female fed on the ground and was later captured in the canopy.

The data presented here may improve local knowledge of ACL epidemiology. This is significant because much of the local populous works in the Amazonian forests [6, 8] where the sylvatic transmission cycle occurs, and therefore these people experience greater exposure to *Leishmania*-infected vectors.

## Conclusions

Our study demonstrates that sand fly abundance and richness is higher in the canopy than at ground level, and these findings corroborate other vertical stratification studies. Certain factors, including the presence of blood meals, most likely influence sand fly distribution between strata. Our findings also corroborate earlier studies conducted in Rondônia State which indicate that *Lu*. *antunesi* and *Lu*. *davisi* play a role as vectors [10, 11, 13, 18, 19]. The fact that these species are present in both strata may indicate that they contribute to parasite transmission between strata. This information augments our knowledge of sand flies in Rondônia and may help improve leishmaniasis surveillance and control programmes.

## Supplementary information

**Additional file 1: Dataset S1.** R scripts.

**Additional file 2: Dataset S2.** R help functions.

## Data Availability

Data supporting the conclusions of this article are included within the article and its additional files. The newly generated sequences were deposited in the GenBank database under the accession numbers MT234139, MT260076-MT7260081, MT293514-MT7293518 and MT300189. Raw data are available without restriction upon request.

## Abbreviations

CL: cutaneous leishmaniasis
LCL: localized lesions (LCL)
ML: mucocutaneous leishmaniasis
DCL: disseminated cutaneous leishmaniasis
ADCL: anergic diffuse cutaneous leishmaniasis
*cytb*: cytochrome *b*
kDNA: kinetoplast DNA
*hsp*70: heat-shock protein 70
MFU: Management Forest Unit
ICMBIO: Instituto Chico Mendes Institute for Biodiversity Conservation
SISBIO: Biodiversity Authorization and Information System
SISGEN: National System of Genetic Heritage Management and Associated Knowledge
PCR: polymerase chain reaction
mtDNA: mitochondrial DNA
EDA: exploratory data analysis
